# Adamantinomatous and papillary craniopharyngiomas are characterized by distinct epigenomic as well as mutational and transcriptomic profiles

**DOI:** 10.1186/s40478-016-0287-6

**Published:** 2016-02-29

**Authors:** Annett Hölsken, Martin Sill, Jessica Merkle, Leonille Schweizer, Michael Buchfelder, Jörg Flitsch, Rudolf Fahlbusch, Markus Metzler, Marcel Kool, Stefan M. Pfister, Andreas von Deimling, David Capper, David T. W. Jones, Rolf Buslei

**Affiliations:** Institute of Neuropathology, Friedrich-Alexander University Erlangen-Nürnberg (FAU), Schwabachanlage 6, D-91054 Erlangen, Germany; German Cancer Consortium (DKTK) and Division of Biostatistics, German Cancer Research Center (DKFZ), Heidelberg, Germany; Department of Neuropathology, Ruprecht-Karls-University Heidelberg, Heidelberg, Germany; Clinical Cooperation Unit Neuropathology, German Cancer Consortium (DKTK), German Cancer Research Center (DKFZ), Heidelberg, Germany; Institute of Neurosurgery, Friedrich-Alexander University Erlangen-Nürnberg (FAU), Erlangen, Germany; Department of Neurosurgery, University Hamburg-Eppendorf, Hamburg, Germany; Department of Neurosurgery, International Neuroscience Institute, Hannover, Germany; Institute of Pediatrics, Friedrich-Alexander University Erlangen-Nürnberg (FAU), Erlangen, Germany; German Cancer Consortium (DKTK) and Division of Pediatric Neurooncology, German Cancer Research Center (DKFZ), Heidelberg, Germany; Department of Pediatric Oncology, Hematology & Immunology, Heidelberg University Hospital, Heidelberg, Germany

**Keywords:** Craniopharyngiomas, *BRAF V600E*, Illumina, β-Catenin, Wnt, SHH

## Abstract

**Introduction:**

Craniopharyngiomas (CP) are rare epithelial tumors of the sellar region. Two subtypes, adamantinomatous (adaCP) and papillary CP (papCP), were previously identified based on histomorphological and epidemiological aspects. Recent data indicates that both variants are defined by specific genetic alterations, and influenced by distinct molecular pathways and particular origins. The fact that CP is an uncommon tumor entity renders studies on large cohorts difficult and exceptional. In order to achieve further insights distinguishing CP variants, we conducted whole genome methylation (450 k array) and microarray-based gene expression studies in addition to *CTNNB1* and *BRAF* mutation analysis using a comprehensive cohort of 80 adaCP and 35 papCP.

**Results:**

*BRAFV600E* mutations were solely found in the papCP subgroup and were not detectable in adaCP samples. In contrast, *CTNNB1* mutations were exclusively detected in adaCP. The methylome fingerprints assigned DNA specimens to entity-specific groups (papCP (*n* = 18); adaCP (*n* = 25)) matching perfectly with histology-based diagnosis, suggesting that they represent truly distinct biological entities. However, we were not able to detect within the adaCP group (including 11 pediatric and 14 adult cases) a significant difference in methylation signature by age. Integrative comparison of the papCP with the adaCP group based on differential gene expression and methylation revealed a distinct upregulation of Wnt- and SHH signaling pathway genes in adaCP.

**Conclusions:**

AdaCP and papCP thus represent distinct tumor subtypes that harbor mutually exclusive gene mutations and methylation patterns, further reflected in differences in gene expression. This study demonstrates that DNA methylation analyses are an additional method to classify CP into subtypes, and implicates a role of epigenetic mechanisms in the genesis of the respective CP variants. Detection of tumor-specific signaling pathway activation enables the possibility of target-oriented intervention.

**Electronic supplementary material:**

The online version of this article (doi:10.1186/s40478-016-0287-6) contains supplementary material, which is available to authorized users.

## Introduction

Craniopharyngiomas (CPs) are defined as histologically benign epithelial tumors of the sellar region [27, 34]. Reported world-wide incidence rates of 1.86 (1.60–2.14) new cases per million per year for all ages and 2.14 (1.53–2.92) for children under 15 years demonstrate that they represent rare lesions [15, 37, 39]. Although the growth pattern of CP is often locally aggressive and treatment is challenging, histological signs of malignancy are missing-thus defining them as grade I tumors according to the World Health Organization (WHO) classification [31]. There are two different subtypes of CP, papillary (papCP) and adamantinomatous (adaCP). Although clear histomorphological differences between both variants exist, a correct diagnosis is sometimes difficult to obtain, especially in small and/or fragmented specimens. Furthermore, the existence of mixed forms and the cell of origin of these tumors are areas of ongoing scientific debate [40, 41].

The papCP variant occurs almost exclusively in adults, at an age of 40–55 years [12]. They are composed of compact, monomorphic sheets of well-differentiated squamous epithelium and typically lack regressive changes like cholesterol clefts, calcifications, wet keratin and inflammation. Ciliated epithelium and PAS+ goblet cells are sometimes encountered, and histological morphology resembles that of Rathke’s cleft cysts with squamous metaplasia.

The adaCP variant is the most common non-neuroepithelial intracerebral neoplasm in children, accounting for 5–11 % of intracranial tumors in this age group [19, 42]. A second peak occurs in adulthood between 50–74 years [7]. Histopathological hallmarks of adaCP are the formation of differentiated epithelium disposed in cords, lobules, nodular whorls and irregular trabeculae bordered by palisaded columnar epithelium. Cystic cavities containing cell debris and fibrosis are lined by flattened epithelium. Pale nodules containing anucleate “ghost cells”/”wet keratin”, large areas of regressive changes i.e. inflammation and calcifications are representative. Further characteristic features of adaCP are finger-like tumor protrusions and the formation of a tumor-specific cellular environment in the surrounding brain tissue [8, 22]. This is accompanied by serious endocrinological and visual disturbances and significant long term morbidity and mortality rates, and makes treatment quite a challenge [26, 36].

Recent data has provided important insights into the molecular pathogenesis and origin of CP, and may provide distinguishing features for both subtypes associated with new molecular drug targets. Today it is common knowledge that the Wnt signaling pathway is strongly implicated in the pathogenesis of adaCP. Genetic analyses have shown that up to 95 % of the tumors harbor activating mutations in exon 3 of the *CTNNB1* gene encoding β-catenin [5, 10, 28, 45]. Genetic alterations within the degradation targeting box of β-catenin lead to activation of the pathway in adaCP, indicated by aberrant nuclear accumulation of the protein and respective Wnt target gene activation [24]. This is described to be exclusive for adaCP [5, 30] and aberrant (nuclear) immunohistochemical staining for β-catenin in whirl-like cell clusters and single cells represent the most reliable marker for adaCP in the differential diagnosis of space occupying lesions in the sellar region [21]. Activated Wnt signaling influences tumor cell migration and the tumor initiating strength of this alteration was recently confirmed *in vivo* [18, 22, 48]. Furthermore, it was shown that EGFR- and SHH signaling pathways are also up-regulated in adaCP and associated with tumor cell migration [1, 2, 20, 23].

The molecular background of papCP initiation was largely unknown until exome sequencing studies revealed *BRAF* mutations (BRAF p.Val600Glu) in up to 95 % of these tumors [5]. This result was confirmed in smaller series of samples, where *BRAF V600E* mutations could be reliably detected by immunohistochemistry [29, 30, 44]. Interestingly, Brastianos and colleagues postulated that *CTNNB1* and *BRAF* mutations were exclusive and clonal in each CP subtype, and they detected no other recurrent mutations or genomic aberrations in either subtype [5, 30]. In contrast, Larkin et al. claimed that *BRAF* mutations may coexist with *CTNNB1* mutations in adamantinomatous tumors [30].

*BRAF* mutations were described in several other neoplasms and potent drugs have already shown a robust clinical response in melanomas, hairy cell leukemias, as well as brain tumors with BRAF p.Val600Glu such as pilocytic astrocytoma, pleomorphic xanthoastrocytoma and ganglioglioma [13, 14, 16, 33, 43]. Recently, two case reports of BRAF inhibitor treatment (Vemurafenib and Dabrafenib in combination with Trametinib) in patients with residual or recurrent papCP after surgery have been published, showing favorable short-term effects. However, the Vemurafenib-treated tumor displayed tumor regrowth after a drug holiday, indicating that further long-term studies are necessary [3, 4].

In order to verify and strengthen the above summarized molecular data and to reveal distinctive molecular characteristics that facilitate the diagnosis of both CP subtypes, we obtained *CTNNB1* and *BRAF* mutational analysis as well as array-based gene expression and methylation profiling in one of the largest cohorts of human CP tissue samples published to date.

## Materials and methods

### Patient cohort

Surgical specimens from patients with CP were retrieved from the archive of the Department of Neuropathology at the University Hospital of Erlangen (*n* = 107) and from the department of Neuropathology at the University of Heidelberg (*n* = 8). The investigated samples contained 35 papillary CP (papCP; 20 female and 15 male patients; mean age = 46.3 years) and 80 adamantinomatous CP (adaCP) from 79 different patients (40 female and 39 male patients; mean age = 35.7 years). The adaCP samples included 25 childhood (mean age of patients ≤16 years = 8.5 years) and 55 adult (mean age = 48.2 years) tumor samples. One adult patient (marked by an asterisk in Table 1) had a relapse 14 years after the first surgery and both tumor samples were selected for mutational analysis. Methylation analysis was performed from 18 papCP and 25 adaCP. AdaCP consisted of 11 childhood and 14 adulthood tumors in order to analyze whether there are differences in the methylation profiles in respective age groups. Gene expression analysis was performed for 10 papCP and 18 adaCP for which good quality RNA was available.

**Table 1:** Summary of mutational and immunohistochemical analyses

Case	Age	Sex	Mutational analysis			Immunohistochemistry	
			*CTNNB1* exon 3	*BRAF* exon 15	*BRAF* Pyro	VE1	nuclear β-catenin
pap1	37	f	*wt*	*V600E*	*V600E*	*+*	neg
pap2	52	m	*wt*	*V600E*	*-*	*-*	neg
pap3	19	m	*wt*	*V600E*	*-*	*-*	neg
pap4^d,e^	49	f	*wt*	*V600E*	*V600E*	*++*	neg
pap5	43	f	*wt*	*V600E*	*-*	*-*	neg
pap6	57	f	*wt*	*V600E*	*-*	*-*	neg
pap7^d,e^	42	m	*wt*	*V600E*	*V600E*	*++*	neg
pap8^d^	46	f	*wt*	*V600E*	*-*	*-*	neg
pap9^d,e^	58	m	*wt*	*V600E*	*V600E*	*++*	neg
pap10	40	m	*wt*	*V600E*	*V600E*	*+*	neg
pap11^b,d,e^	38	m	*wt*	*V600E*	*-*	*-*	neg
pap12	57	m	*wt*	*V600E*	*-*	*-*	neg
pap13^d,e^	39	m	*wt*	*V600E*	*V600E*	*++*	neg
pap14	61	f	*wt*	*V600E*	*-*	*-*	-
pap15	56	f	*wt*	*V600E*	*-*	*-*	neg
pap16^d,e^	45	m	*wt*	*V600E*	*-*	*-*	neg
pap17	62	f	*wt*	*V600E*	*-*	*-*	neg
pap18^d,e^	36	m	*wt*	*V600E*	*V600E*	*+*	neg
pap19^d,e^	40	m	*wt*	*V600E*	*V600E*	*-*	neg
pap20^d^	36	f	*wt*	*V600E*	*-*	*-*	neg
pap21^e^	48	f	*wt*	*V600E*	*V600E*	*+*	neg
pap22^e^	48	f	*-*	*-*	*V600E*	*++*	neg
pap23^e^	34	m	*wt*	*V600E*	*V600E*	*++*	neg
pap24	43	m	*wt*	*V600E*	*-*	*-*	neg
pap25	41	f	*wt*	*V600E*	*-*	*-*	neg
pap26	47	f	*wt*	*V600E*	*V600E*	*-*	neg
pap27	34	f	*wt*	*V600E*	*-*	*-*	neg
pap28	74	m	*wt*	*V600E*	*V600E*	*-*	neg
pap29^a,e^	53	f	*-*	*-*	*-*	*-*	-
pap30^a,e^	41	f	*-*	*-*	*-*	*-*	-
pap31^a,e^	51	f	*wt*	*-*	*V600E*	*-*	-
pap32^a 2^	50	m	*wt*	*-*	*V600E*	*-*	-
pap33^a,e^	23	f	*wt*	*-*	*V600E*	*-*	-
pap34^a,e^	69	f	*wt*	*-*	*V600E*	*-*	-
pap35^a,e^	50	f	*-*	*-*	*V600E*	*-*	-
ada1	33	m	*S33C*	*wt*	*-*	*-*	neg
ada2	26	f	*S33P*	*wt*	*-*	*-*	pos
ada3	33	f	*G34V*	*wt*	*-*	*-*	pos
ada4	42	m	*S33A; S33C*	*wt*	*-*	*-*	pos
ada5	14	f	*S37Y*	*wt*	*-*	*-*	pos
ada6^d,e^	14	m	*T41I*	*wt*	*-*	*-*	pos
ada7^c^	61	f	*S45P*	*wt*	*-*	*-*	neg
ada8^c^	47	f	*S45P*	*wt*	*-*	*-*	pos
ada9	2	f	*T41I*	*wt*	*-*	*-*	pos
ada10	3	m	*D32H*	*wt*	*-*	*-*	pos
ada11^d,e^	11	m	*T41I*	*wt*	*wt*	*-*	pos
ada12	64	f	*S45F*	*wt*	*-*	*-*	pos
ada13	77	m	*S33A*	*wt*	*-*	*-*	-
ada14	20	m	*A32F*	*wt*	*-*	*-*	pos
ada15	49	m	*G34R*	*wt*	*-*	*-*	pos
ada16^d,e^	56	m	*S33F*	*wt*	*wt*	*-*	pos
ada17	21	f	*S33Y*	*wt*	*-*	*-*	pos
ada18	70	m	*S33C*	*wt*	*-*	*-*	pos
ada19^d,e^	9	f	*S33C; T41S*	*wt*	*-*	*-*	pos
ada20	8	m	*S33C*	*wt*	*-*	*-*	pos
ada21^d,e^	15	m	*T41I*	*wt*	*-*	*-*	pos
ada22	54	f	*S33F*	*wt*	*-*	*-*	pos
ada23	4	f	*S37F*	*wt*	*-*	*-*	pos
ada24	52	f	*G34C*	*wt*	*wt*	*-*	pos
ada25	51	m	*G34R*	*wt*	*-*	*-*	pos
ada26	47	f	*T41I*	*wt*	*-*	*-*	pos
ada27	43	f	*G34V*	*wt*	*-*	*-*	neg
ada28	48	f	*T41I*	*wt*	*-*	*-*	pos
ada29^d,e^	66	m	*T41I*	*wt*	*wt*	*-*	pos
ada30^d^	11	f	*T41A*	*wt*	*wt*	*-*	pos
ada31^d,e^	7	m	*G34R*	*wt*	*-*	*-*	pos
ada32^d,e^	50	m	*I35S*	*wt*	*wt*	*-*	pos
ada33^d,e^	15	f	*T41A*	*wt*	*wt*	*-*	pos
ada34	6	f	*I35I; S37F*	*wt*	*-*	*-*	pos
ada35	57	f	*D32Y*	*wt*	*-*	*-*	pos
ada36	58	f	*S33C*	*wt*	*-*	*-*	pos
ada37^d,e^	32	m	*D32V*	*wt*	*-*	*-*	pos
ada38	28	m	*D32N*	*wt*	*-*	*-*	pos
ada39	6	m	*G34V*	*wt*	*-*	*-*	pos
ada40	66	f	*S37C*	*wt*	*-*	*-*	pos
ada41	48	m	*S37F*	*wt*	*-*	*-*	-
ada42	67	m	*G34R*	*wt*	*-*	*-*	pos
ada43	3	f	*S37F*	*wt*	*wt*	*-*	pos
ada44	47	f	*S33C*	*wt*	*-*	*-*	pos
ada45	64	m	*S33C*	*wt*	*-*	*-*	pos
ada46^e^	3	m	*S33F*	*wt*	*wt*	*-*	pos
ada47	5	f	*G34V*	*wt*	*-*	*-*	pos
ada48^d,e^	59	m	*S33C*	*wt*	*wt*	*-*	pos
ada49	72	m	*S37V*	*wt*	*-*	*-*	pos
ada50	4	f	*D32H*	*wt*	*-*	*-*	pos
ada51	53	m	*S33C*	*wt*	*-*	*-*	-
ada52	45	m	*T41I*	*wt*	*-*	*-*	pos
ada53^d,e^	38	f	*S33C*	*wt*	*wt*	*-*	pos
ada54^d,e^	16	f	*D32N*	*wt*	*wt*	*-*	pos
ada55	38	f	*T41I*	*wt*	*-*	*-*	pos
ada56^d,e^	39	m	*S33C*	*wt*	*wt*	*-*	pos
ada57	61	f	*S33C*	*wt*	*-*	*-*	pos
ada58^d,e^	58	m	*S33A*	*wt*	*wt*	*-*	pos
ada59^d,e^	45	m	*36_37del*	*wt*	*wt*	*-*	pos
ada60^e^	48	m	*D32Y*	*wt*	*-*	*-*	pos
ada61	28	m	*S33C*	*wt*	*-*	*-*	pos
ada62	64	f	*T41A*	*wt*	*-*	*-*	-
ada63	53	f	*S33C*	*wt*	*-*	*-*	pos
ada64	9	m	*S33F*	*wt*	*-*	*-*	pos
ada65	8	f	*S33C*	*wt*	*-*	*-*	pos
ada66^e^	50	f	*D32H*	*wt*	*wt*	*-*	pos
ada67	20	f	*T41I*	*wt*	*-*	*-*	pos
ada68	50	m	*S33C*	*wt*	*-*	*-*	pos
ada69	25	f	*D32H*	*wt*	*-*	*-*	pos
ada70^d,e^	37	m	*D32H*	*wt*	*wt*	*-*	pos
ada71	12	f	*T41I*	*wt*	*wt*	*-*	pos
ada72	54	m	*del-85bp*	*wt*	*-*	*-*	pos
ada73	27	f	*S33C*	*wt*	*-*	*-*	pos
ada74	61	f	*D32F*	*wt*	*-*	*-*	pos
ada75^e^	37	m	*35-46del*	*wt*	*wt*	*-*	pos
ada76^e^	51	m	*D32Y*	*wt*	*wt*	*-*	pos
ada77	63	m	*S33F*	*wt*	*-*	*-*	pos
ada78^e^	9	f	*-*	*-*	*-*	*-*	pos
ada79^e^	9	f	*S33F*	*-*	*wt*	*-*	pos
ada80^a e^	9	f	*T41A*	*-*	*wt*	*-*	-

Age illustrates time at tumor surgery. Samples were screened for *CTNNB1* and *BRAF* mutation using different standard techniques

*VE1* BRAF V600E specific antibody, *m* male, *f* female, *wt* wild type, *pos* positive, *neg* negative, *Pyro* Pyrosequencing

^a^Patient cohort under study comprising tissue specimens of papillary (pap) and adamantinomatous (ada) craniopharyngiomas (CP), collected from the archives of the Departments of Neuropathology in Erlangen and Heidelberg

^b^ = unclear CP subtype, − = not analysed, ^c^ = samples of one patient with a recurrent tumor after 14 years

^d^samples subject to analyses of gene expression using Affymetrix U133 Plus2.0 expression array or ^e^450 k Illumina methylation array

Each tumor sample was classified according to World Health Organization (WHO) guidelines using haematoxylin and eosin as well as immunohistochemical stainings e.g. pan-cytokeratin (KL-1) and β-catenin. We included one CP (pap11) where subtype specification was initially difficult to obtain by histological and immunohistochemical criteria. A declaration of consent of each patient is available for all specimens for further scientific investigation, approved by the local ethics committee of the University of Erlangen. Procedures were conducted in accordance with the Declaration of Helsinki.

### Immunohistochemistry

Surgical samples were prepared as previously described [9]. The slides were stained using a Ventana BenchMark ultraimmunostainer (Ventana, Tuscon, AZ, USA) and the following antibodies: monoclonal mouse-anti-β-catenin (1:800, Clone 14, BD Biosciences, Franklin Lakes, New Jersey); monoclonal antibody that selectively recognizes the BRAF V600E mutant epitope (BRAF V600E-specific clone VE1, Ventana, USA). The staining protocol included pretreatment with cell conditioner 1 (pH 8,4) for 64 min, incubation with antibody at 36 °C for 16 minutes, primary antibody detection using the ultraView Universal DAB Detection Kit (Ventana), followed by counterstaining with hematoxylin for 4 minutes. Validation of the VE1 antibody has been previously reported in detail [11].

### DNA and RNA preparation

We selected representative native tumor samples and paraffin embedded tissue for mutational analysis and confirmed vital tumor content microscopically in each case. DNA was extracted using the DNeasy tissue kit and the QIAamp DNA micro kit purchased from Qiagen (Hilden, Germany). Pooled DNA obtained from peripheral blood leukocytes of healthy persons were extracted with the Blood DNA kit (Qiagen) according to the manufacturer’s instructions and served as wild type controls. RNA from snap frozen tissue was isolated with the RNeasy extraction kit (Qiagen) followed by subsequent digestion with RNase-free DNase I and purification via RNeasy columns (Qiagen).

DNA and RNA concentration was determined using the Qubit® dsDNA HS Assay Kit or RNA Assay Kit (Life Technologies, Eugene, USA).

### Mutational analysis

Genomic DNA of exon 3 of *CTNNB1* (encoding β-catenin) and exon 15 of *BRAF* was amplified for single strand conformation polymorphism (SSCP) analysis using the following primer pairs: *CTNNB1*_for: GATTTGATGGAGTTGGACATGG; *CTNNB1*_rev: TGTTCTTGAGTGAAGGACTGAG (218 bp); or *CTNNB1*_for: AGTTGGACATGGCCATGGAA; *CTNNB1*_rev: ACATCCTCTTCCTCAGGATT (145 bp); *BRAF*_for: TCCTTTACTTACTACACCTCA; *BRAF*_rev: AGTAACTCAGCAGCATCTCA (204 bp). PCR reactions were performed in a total volume of 10 μl with at least 10 ng genomic DNA. The amplified PCR products were denatured for 10 min at 94 °C. SSCP electrophoresis of exons and exon fragments was performed on polyacrylamide gels (14 %) and a bis-acrylamide/acrylamide ratio of 1:99, with 5 % glycerol in 0.5x TBE at room temperature. The single and double strands of the PCR products were visualized by silver staining, as described previously [6]. The shifted bands were excised from the wet gel, eluted and reamplified. The purified PCR-products were sent to GATC Biotech AG (Konstanz, Germany) for DNA sequencing. *CTNNB1* mutation analysis was conducted as described in detail elsewhere [10].

### Pyrosequencing

Pyrosequencing of BRAF codon 600 was carried out with the therascreen® BRAF Pyro Kit (Qiagen, cat.no 971470) on the PyroMark Q24 platform (Qiagen) according to manufacturer’s instructions. Pyrograms were generated and analyzed with the PyroMark Q24 software (version 2.0.6.). The light signal (in relative light/fluorescent units, RLU) generated by the pyrosequencing reactions is proportional to the amount of DNA template and the number of nucleotides incorporated into a target DNA strand. Data are displayed in the form of a pyrogram, a series of peaks on a graph. The height ratio between the peaks allows for the estimation of allele frequencies (in % units). The mutant allele frequency is the proportion of a particular variant allele among all allelic copies of a sample. Samples with a mutation frequency greater than the limit of detection (LOD) plus 3 % units were scored as mutation positive. Evaluation of potential low level mutations (mutation frequency ≥ LOD and ≤ LOD + 3 % units) was performed taking into account histological tumor cell content and VE1 immunohistochemical findings.

### Gene expression profiling

Affymetrix U133 Plus2.0 expression array data were generated at the Microarray Department of the University of Amsterdam, the Netherlands according to manufacturer’s instructions. The MAS5.0 algorithm of the GCOS program (Affymetrix Inc) was used for normalization and assignment of detection *p*-values. Array quality was ensured by inspection of beta-actin and GAPDH 5′-3′ ratios as well as the percentage of present calls. Data were further interrogated using the R2 microarray analysis suite (http://r2.amc.nl). For this analysis 18 adaCP and 10 papCP samples (Table 1) were included.

To cluster samples and determine an optimal number of stable clusters, unsupervised consensus clustering [35] was applied. Clustering was performed using the log2 gene expression values of the 5000 most variably expressed genes as measured by standard deviation. Within each of the 1000 resampling iterations of the consensus clustering, a hierarchical clustering using ward linkage as agglomeration method and euclidean distance as distance measure was applied. The most stable clustering for K = 2 clusters was determined by visually inspecting the resulting consensus matrices and delta-K criteria.

### Methylome profiling

For this analysis DNA from snap frozen tissue (*n* = 10) or formalin-fixed tissue (*n* = 33) with tumor content >60 % was extracted. The Illumina Infinium HumanMethylation450 (450 k) array was used to obtain the DNA methylation status of 482,421 CpG sites (Illumina, San Diego, USA), according to the manufacturer’s instructions at the Genomics and Proteomics Core Facility of the DKFZ. The methylation level of each CpG site was represented by beta-values, which ranged from 0 (unmethylated) to 1 (methylated). The following criteria were applied to filter the data: removal of probes targeting sex chromosomes, removal of probes containing a single nucleotide polymorphism (dbSNP132 Common) within five base pairs of and including the targeted CpG-site (*n* = 24,536), and probes not mapping uniquely to the human reference genome (hg19) allowing for one mismatch (*n* = 9993). In total, 438,370 probes were kept for analysis.

To cluster samples and determine an optimal number of stable clusters, unsupervised consensus clustering [35] was applied. Clustering was performed using the beta values of the 10,000 most variably methylated probes as measured by median absolute deviation (MAD). Within each of the 1000 resampling iterations of the consensus clustering, a hierarchical clustering using ward linkage as agglomeration method and euclidean distance as distance measure was applied. The most stable clustering for K = 2 clusters was determined by visually inspecting the resulting consensus matrices and delta-K criteria.

To reorder the 10,000 most variable probes for the heatmap visualization, probes were clustered by agglomerative hierarchical clustering using the euclidean distance as distance measure and average linkage as agglomeration method.

In addition, to apply a different unsupervised method to reveal groups of samples within the methylation data, principle component analysis (PCA) was applied to the same 10,000 probes selected for the clustering, but transformed to M-values by logit-transformation before applying PCA.

A scatterplot that shows the samples projected onto the first two principle components is shown in Fig. 2a.

### Differential gene expression and methylation analysis

Ensemble gene annotations (GRCh37.p5) and Affymetrix probe localizations were obtained from MartView (http://www.biomart.org). For the integrated analysis only cases for which both methylation and gene expression data were available were used (*n* = 25). The cohort includes samples of eight papCP and 17 adaCP (Table 1). CpG sites were mapped to genes by aggregating the methylation beta values in the range of + − 5 kb of the transcription start site (TSS) of all protein-coding transcripts by taking the mean of the 25 % most variant CpG probes measured by MAD. After excluding non-protein-coding transcripts and genes with no measured CpG sites in the considered region around the TSS, the total number of genes was 12,548. T-tests were applied to identify genes which are differentially methylated or differentially expressed between adaCP and papCP. To meet the normality assumption of the t-test, aggregated beta methylation values were transformed to M-values by logit-transformation. In addition, to deal with the multiple testing problem the Bonferroni correction was applied and genes with a *p*-value < α/(2×12,548) were considered significantly, differentially expressed/methylated. The nominal type one error level to control the family-wise error rate (FWER) was α = 5 %. In addition, to find age-dependent differences, 7 childhood and 10 adult adaCP samples were compared by following the same procedure.

## Results

### AdaCP and papCP harbor distinctive gene mutations

Mutational analysis of *CTNNB1 and BRAF* was performed in 110 and 112 human CP tumor samples respectively (Table 1). Genetic alterations within the *CTNNB1* gene were analyzed using SSCP followed by direct sequencing of divergent bands. We found activating mutations and small deletions affecting exon 3 of *CTNNB1* exclusively in the group of adaCP. Most of the identified aberrations directly involved serine or threonine phosphorylation sites of β-catenin, being essential for its degradation and inhibition [Codon 33 in 28 cases, followed by codons 41 (*n* = 16) and 32 (*n* = 13)]. In accordance with this, we were able to detect tumor cells with nuclear β-catenin accumulations in 96 % (72/75) of the adaCP specimens but in none of the papCP samples examined. The results are summarized in Table 1.

**Fig. 1:**
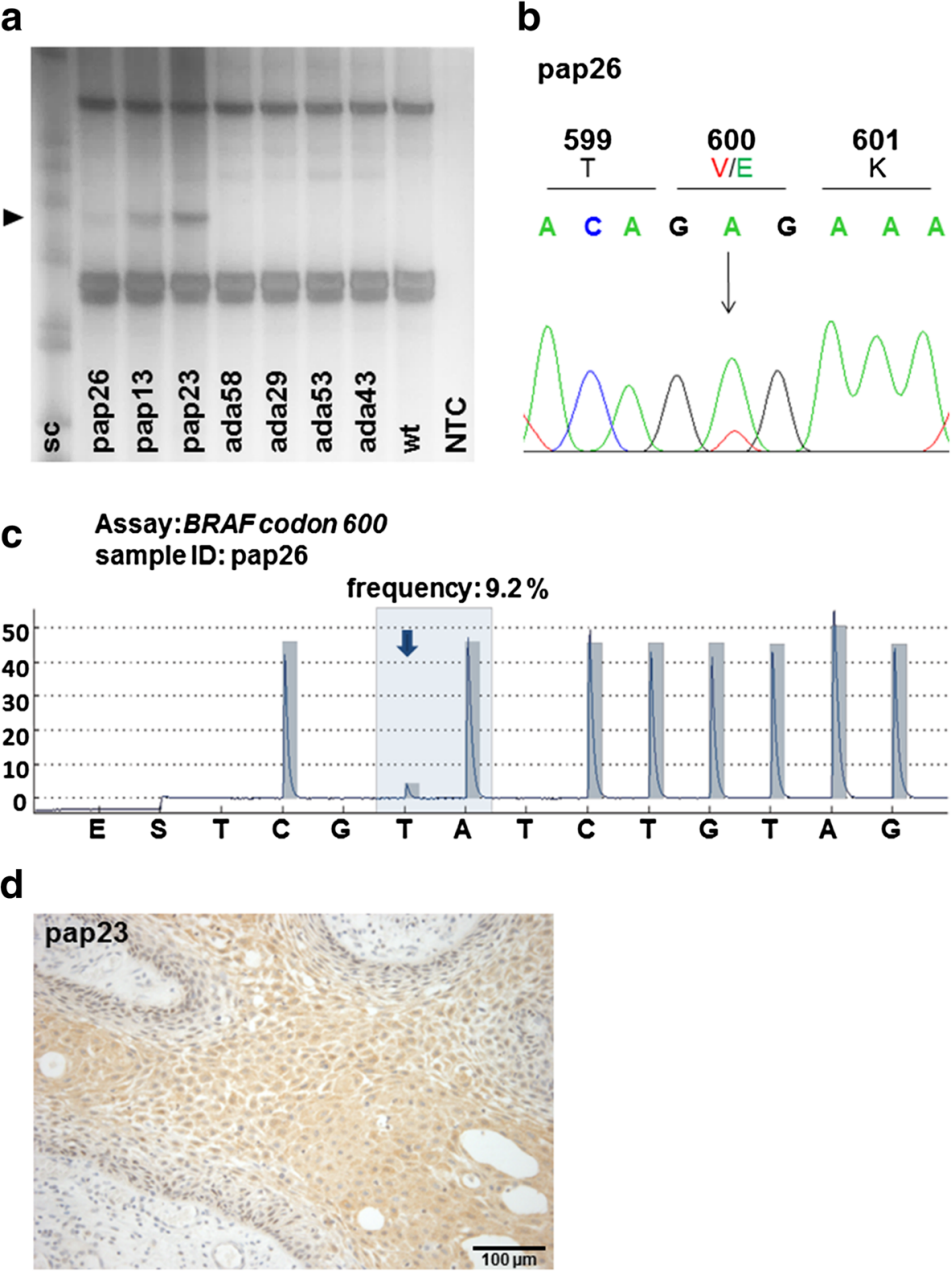
Detection of *BRAF V600E* mutation in papCP. SSCP analyses revealed shifted bands (▸) only in papCPs (**a**). DNA extraction and subsequent Sanger sequencing confirmed a *BRAF V600E* mutation (**b**). Pyrosequencing was utilized in cases with only slight SSCP bands or less available DNA concentrations. Case pap26 exhibited a low (9.2 %) frequency of BRAFV600E mutation (**c**). Immunohistochemical staining using a mutation specific BRAF V600E antibody (clone VE1) revealed a positive staining of pap23 rated with ++ (**d**). sc = staining control; wt = wild type; NTC = non template control

*BRAF* mutations were only detectable in papCP (33/33) but not in the large group of adaCP (0/79) examined in our series. SSCP analysis displayed shifted bands of variable intensity in all of the analyzed (*n* = 27) papCP tumor samples (Fig. 1a). Subsequent Sanger sequencing confirmed the presence of *BRAF V600E* mutation (Fig. 1b) and additional pyrosequencing of 18 papCP cases revealed (*n* = 6) or verified (*n* = 12) mutations (Fig. 1c, Table 1). In contrast, we were not able to detect any evidence of a *BRAF V600E* mutation in 22 adaCP cases using pyrosequencing. One primary and recurrent adaCP tumor sample from the same patient was available for targeted genotyping and exhibited the same *CTNNB1* mutation (S45P) but no additional alteration.

VE1 immunohistochemistry indicated the presence of mutant BRAF protein in all papCP cases tested (*n* = 10). The staining appeared cytoplasmic with a diffuse to finely granulated, either homogenous or patchy distribution pattern. Staining intensity ranged from weak (+) to moderate (++) (Fig. 1d and Table 1). The labeling in multilayered squamous epithelium was accentuated in the apical zone and in some cases almost undetectable in the basal cell layer.

### Gene expression analysis confirmed over-activation of specific markers and important embryonic signaling pathways in human adaCP

To unravel possible differences in the gene expression profile of human CP subtypes we performed microarray analysis of 18 adaCP and 10 papCP samples. Data analysis revealed significant up-regulation of several direct targets of the Wnt/β-catenin signaling pathway in adaCP compared to papCP, including *LEF1* and *AXIN2* (Additional file 1: Figure S1). Furthermore, important components of the hedgehog signaling pathway e.g. *GLI2*, *PTCH1* as well as *SHH* (Additional file 1: Figure S1) were also over-expressed specifically in adaCP. Gene expression data showed increased expression of MAP2 (*MAP2*), Tenascin C (*TNC*) and the stem cell marker CD133 (*PROM1*) in adaCP (Additional file 1: Figure S1A). *CD44* and Claudin 1 (*CLDN1*), a recently described distinguishing marker for the two variants [47], are significantly down regulated in adaCP (Additional file 1: Figure S1). Values of the gene expression analysis are given in detail for each sample in Additional file 2: Table S1.

Furthermore, unsupervised consensus clustering of the gene expression values of the 5000 most variable genes resulted in two distinct and stable clusters that perfectly separate adaCP and papCP samples.

### DNA methylation profiles differ in adaCP and papCP

**Fig. 2:**
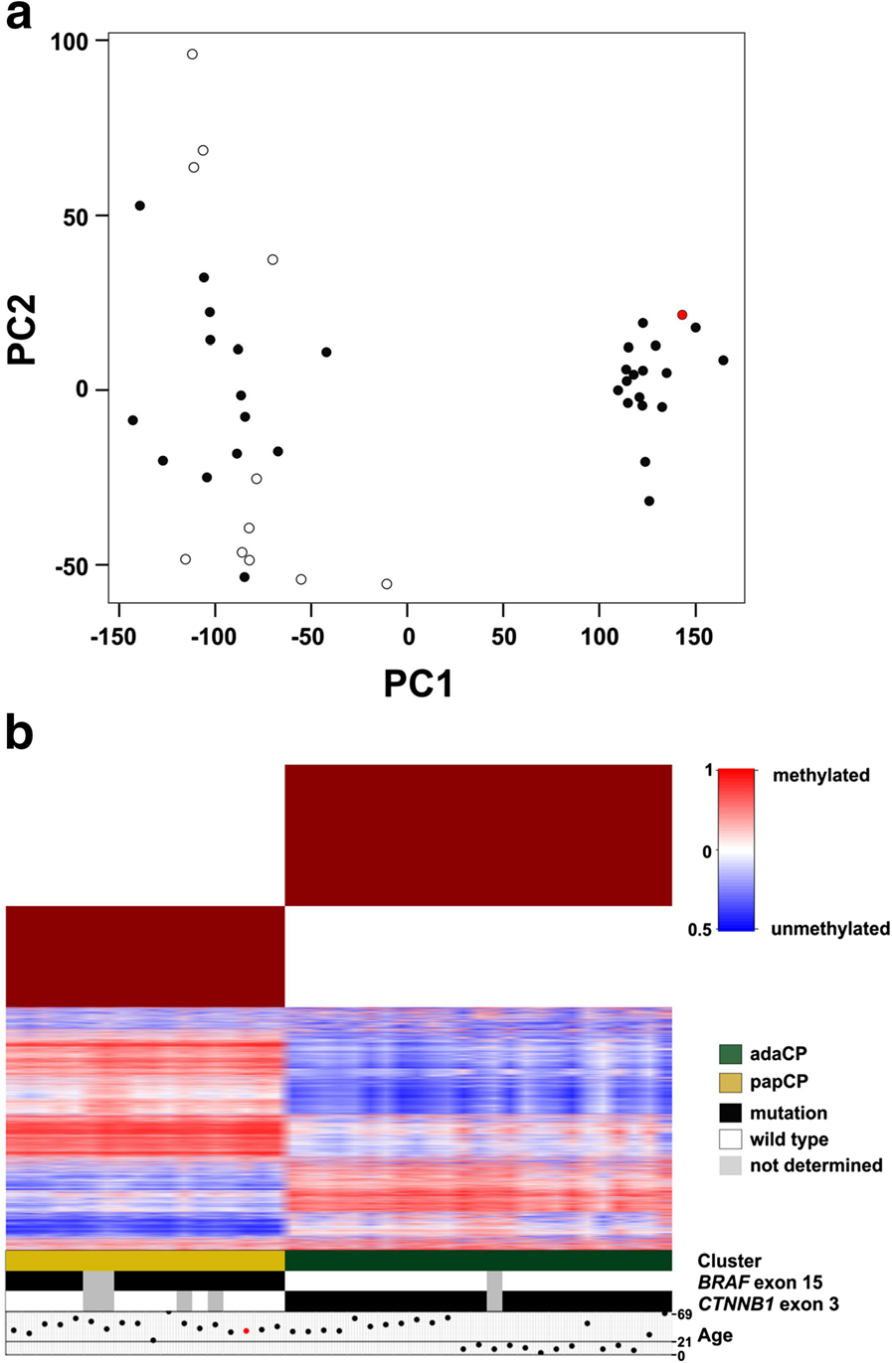
Methylation profiling of CP subtypes. The scatterplot (**a**) shows samples projected onto the first two principle components derived by applying PCA to the most variable 10,000 probes already selected for the clustering. Pediatric CP are marked with white circles and adult CP with black circles. Unsupervised consensus clustering of 450 k methylation data revealed two distinct and stable clusters corresponding to papCP and adaCP, respectively. The upper part of the figure shows the consensus matrix (brown) that displays the stability of the clusters, i.e. in all of the 1, 000 resampling iterations of the consensus clustering the same samples were assigned to the same two clusters (**b**). The lower part of the figure shows a heatmap of the methylation pattern of the 10,000 most variable CpG sites used for clustering. Below the heatmap the two clusters resulting from the consensus clustering of gene expression data is shown. Furthermore, the distribution of age, BRAF and CTNNB1 mutations across samples was added. The sample marked with a red dot represents a case with histologically unsure subtype classification

We then analyzed the most variably methylated CpG sites (10,000 genomic loci) in a cohort of 25 adaCP (11 pediatric and 14 adult cases) and 18 papCP samples (Table 1). Unsupervised hierarchical clustering of these loci revealed two distinct and stable methylation clusters (Fig. 2). Applying PCA to the same CpG sites revealed comparable results, i.e. Fig. 2a shows a scatterplot of the samples projected onto the first two principle components. As with the clustering result of the gene expression data, the first cluster contained only papCP and the second cluster was composed exclusively of adaCP. The histologically unclear case marked in red grouped clearly to the papCP cluster (Fig. 2a, b).

**Fig. 3:**
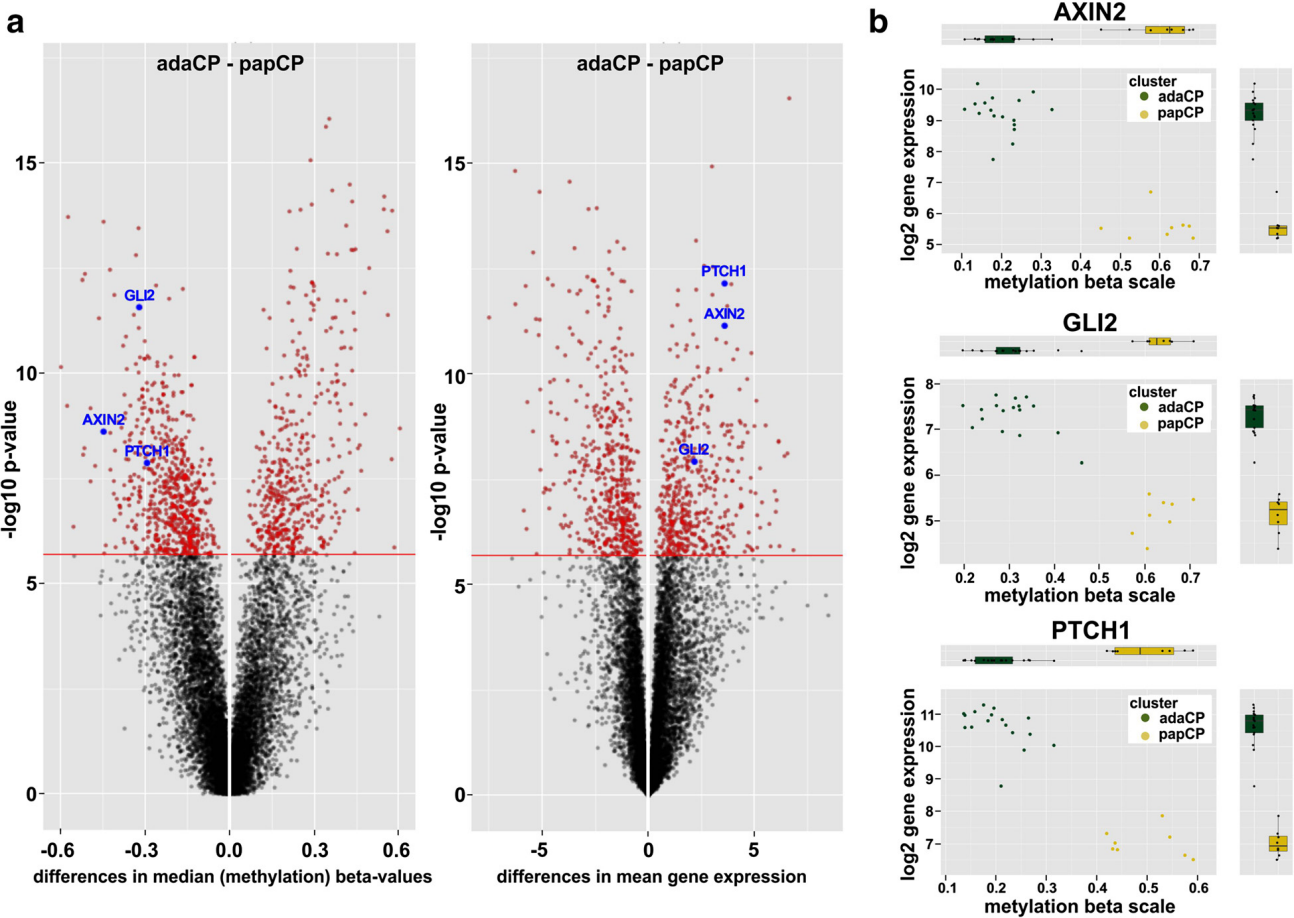
Comparison of adaCP and papCP methylation and gene expression profile. Volcano plot (**a**) showing the difference in median methylation of genes between adaCP (*n* = 17) and papCP (*n* = 8) samples on the x-axis and the –log10 transformed *p*-values of the corresponding t-test results on the y-axis. The volcano plot on the right hand side shows the difference in mean gene expression of genes between adaCP and papCP samples on the x-axis and corresponding –log10 transformed t-test p-values on the y-axis. Genes significantly differentially methylated and differentially expressed are marked red. *AXIN2*, *PTCH1* and *GLI2*, indicated in blue, are significantly hypomethylated and show an increased gene expression in adaCP samples. (**b**) Plotting of tumor specific *AXIN2*, *GLI2* and *PTCH1* methylation values (beta scale, x-axis) and gene expression values (log2, y-axis) revealed a significant different clustering of adaCP and papCP subtypes. Corresponding box plots clearly emphasize that there is an inverse correlation between gene expression and methylation

It is of note, that integrated methylation and gene expression analysis revealed significant hypomethylation of *AXIN2* (Wnt pathway) as well as *GLI2* and *PTCH1* (SHH pathway) associated with high gene expression within the respective tumors (Fig. 3a, b).

However, comparison of the pediatric and adult cases of adaCP group revealed no significant difference in the integrated methylation and gene expression analysis (Additional file 3: Figure S2).

## Discussion

Two different subtypes of craniopharyngiomas (CP) have to be distinguished according to the current version of the WHO classification of tumors of the central nervous system [31]. Both variants, the adamantinomatous CP (adaCP) and the papillary CP (papCP), differ in their histomorphology, age distribution and clinical course. Whereas papCP occur almost exclusively in adults, adaCP have a bimodal age distribution with incidence peaks in children and adults aged 45–60 years [32]. The most significant factor associated with recurrence is the extent of surgical resection which depends on tumor size and localization. Nowadays there is a trend towards less radical extirpation in order to avoid hypothalamic injury [38]. To prevent a higher recurrence rate after incomplete surgical resection, additional radiotherapy is widely used. Histological evidence of brain invasion through the building of finger-like tumor protrusion, which is more frequently documented in the adaCP type than in the papCP type, seems not to correlate with higher recurrence rates in cases with gross surgical resection [49]. Discrimination of CP variants is often challenging in only small and/or fragmented surgical specimens, and the existence of CP with a mixed histological pattern was specified and promoted in several reports [40, 41]. Newly described molecular markers may help to solve this problem, and innovative approaches with large numbers and well characterized tumor samples are required in order to determine important implications for the differential diagnosis and treatment of CP. Our results, obtained in one of the largest cohorts to date, indicate that both variants can clearly be distinguished using genetic and epigenetic profiling. Targeted genotyping revealed activating mutations and small deletions in exon 3 of *CTNNB1* (β-catenin) exclusively in adaCP cases. Using immunohistochemistry we were able to detect tumor cells with nuclear β-catenin accumulation only in the adaCP specimens tested. In contrast, *BRAF* mutations were detectable in all of the papCP tumor samples. Results of SSCP analyses could be verified using pyrosequencing and VE1 immunohistochemistry, with all mutations being of the hotspot *V600E* type. Our results are in line with a previous study published by Brastianos et al., showing *CTNNB1* alterations in 92–96 % of adaCP and *BRAFV600E* mutations in a frequency of 95–100 % of papCP, depending on the detection method and the tumor purity [5]. The assumption that both mutations were mutually exclusive was recently questioned, however. In a small cohort of adaCP two tumors with *CTNNB1* alterations in codon 41 (T41I) and additional *BRAF V600E* mutations were described [30]. To assess this specific correlation we included 11 adaCP with the described *CTNNB1* gene mutation (codon 41, T41I) in our analysis. However, we were not able to detect additional alterations in *BRAF* in any of the adaCP samples studied. We also analyzed one patient showing a tumor relapse occurring 14 years after first surgery, but did not detect additional genomic alterations. Both the primary tumor and recurrence showed the same *CTNNB1* mutation. Our work validates the hypothesis that these mutations are mutually exclusive in the CP subtypes and represent valuable molecular markers to differentiate papCP and adaCP. Several other studies have verified the benefit of *CTNNB1* and *BRAFV600E* in the differential diagnosis of sellar lesions and we support this statement, with the caveat that V600E mutations may also occur for example in sellar pilocytic astrocytomas, and thus supportive histology is also required [21, 29, 44].

The apparent mutual exclusivity of *CTNNB1* and *BRAFV600E* mutations in the CP variants indicates that both subtypes have a different molecular origin. This suggestion is supported by previous studies providing important insights into the molecular and cellular pathogenesis of these tumors [2, 8, 17, 25]. Several pathways have been described to be activated in adaCP cell clusters with nuclear β-catenin accumulations, including the Wnt pathway, the epidermal growth factor receptor pathway and the sonic hedgehog pathway [1, 18, 20, 23, 24, 46]. A novel tumor stem cell niche (CD133 and CD44), a specific cellular environment at the brain invasion border (TNC and MAP2) and a novel role for pituitary stem cells in the pathogenesis of adaCP has been proposed [2, 8, 17, 25]. Using array-based gene expression profiling we were able to confirm these associations in terms of transcriptional differences. Comparing papCP and adaCP tumor samples, the latter showed significant up-regulation of direct targets of the Wnt/β-catenin signaling pathway (*LEF1* and *AXIN2*) as well as important components of the hedgehog signaling pathway (*GLI2*, *PTCH1* and *SHH*). Furthermore, *CLDN1* (a recently introduced marker to differentiate between CP subtypes) showed significantly higher expression levels in papCP [47]. Detection of tumor-specific signaling pathway activation enables the possibility of target-oriented intervention. Testing of novel pharmacological treatments for both CP variants is on the way, and will hopefully provide additional therapeutic options in the future, with BRAF V600E inhibition being a particularly attractive approach.

Reports regarding epigenomic differences in CP are missing so far. Therefore, we conducted DNA methylation profiling and were able to clearly distinguish the groups of adaCP and papCP. This was confirmed by gene expression analysis, demonstrating that the two subtypes are clearly molecularly distinct on both the epigenetic and transcriptional level.

One small biopsy of a histologically challenging case (pap11) was integrated in the analysis and grouped clearly into the papCP cluster, highlighting the utility of this approach for classification. It is of note, that differential methylation analysis revealed no significant difference between adaCP in children and adults. However, differential methylation and gene expression analysis emphasize an epigenetic impact on the Wnt- and Hedgehog signaling in adaCP as a whole. Further studies are currently underway to verify and specifically analyse additional differentially methylated genes and to correlate this data with gene expression profiles and clinical data.

## Conclusion

This study clearly supports the assumption that adaCP and papCP are two distinct entities with different genetic and epigenetic backgrounds. They can be clearly diagnosed in most cases by using *BRAF V600E* and *CTNNB1* mutation analysis but also by their methylation profile.

Innovative approaches studying large and well characterized tumor samples may help to further understand the pathogenesis of both variants and may lead to find new treatment strategies.

## Additional files

Additional file 1: Figure S1. CP subtypes show significant differences in their gene expression profiles. Affymetrix U133 Plus2.0 expression array data (in log2 expression units) of eighteen adaCP and ten papCP illustrated differentially expressed genes involved in Wnt- (*LEF1* and *AXIN2*), Hedgehog signaling (*PTCH1*, *GLI2* and *SHH*), and stem cell characteristics (*TNC, MAP2, PROM1, CD44*) as well as intercellular adhesion (*CLDN1*). (JPG 390 kb) Additional file 2: Table S1. Single Affymetrix U133 Plus2.0 expression array data of all adaCP and papCP analysed, given in log2 expression units. (DOC 22.6 kb) Additional file 3: Figure S2. Pediatric and adult adaCP do not have different methylation or gene expression signatures. Volcano plot (**a**) showing the difference in median methylation of genes between pediatric (*n* = 7) and adult (*n* = 10) adaCP samples on the x-axis and the –log10 transformed p-values of the corresponding t-test results on the y-axis. The volcano plot on the right hand side (**b**) shows the difference in mean gene expression of genes between pediatric and adult adaCP samples on the x-axis and corresponding –log10 transformed t-test *p*-values on the y-axis. No gene was found to be significantly differentially methylated or differentially expressed. (JPG 338 kb)
