# Supplementary figures and images for: Adamantinomatous and papillary craniopharyngiomas are characterized by distinct epigenomic as well as mutational and transcriptomic profiles
Source: Acta Neuropathol Commun. 2016 Feb 29;4:20. doi: 10.1186/s40478-016-0287-6 (PMC4770705; doi:10.1186/s40478-016-0287-6)

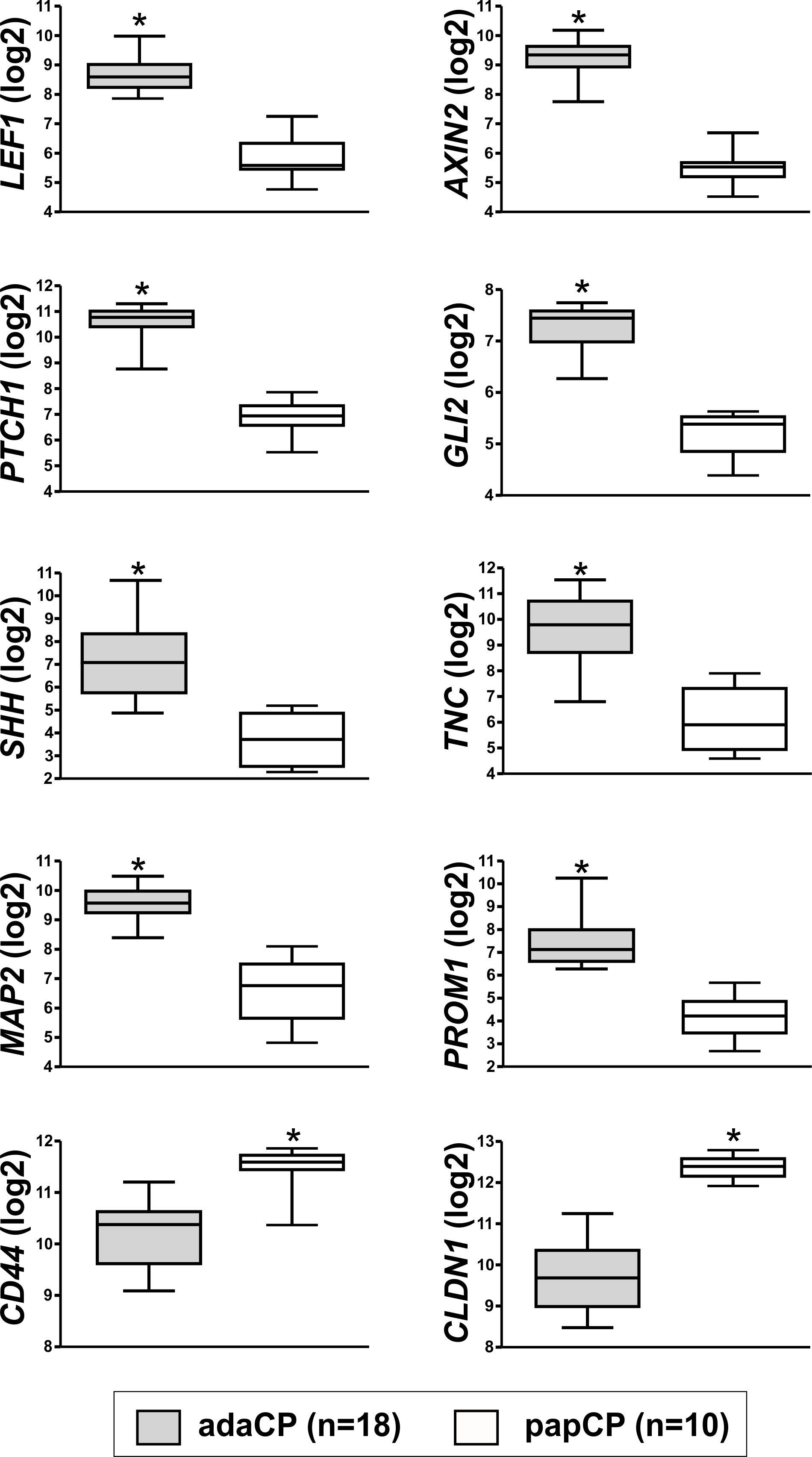

Supplement: Additional file 1: Figure S1. — CP subtypes show significant differences in their gene expression profiles. Affymetrix U133 Plus2.0 expression array data (in log2 expression units) of eighteen adaCP and ten papCP illustrated differentially expressed genes involved in Wnt- (LEF1 and AXIN2), Hedgehog signaling (PTCH1, GLI2 and SHH), and stem cell characteristics (TNC, MAP2, PROM1, CD44) as well as intercellular adhesion (CLDN1). (JPG 390 kb) [file 40478_2016_287_MOESM1_ESM.jpg]

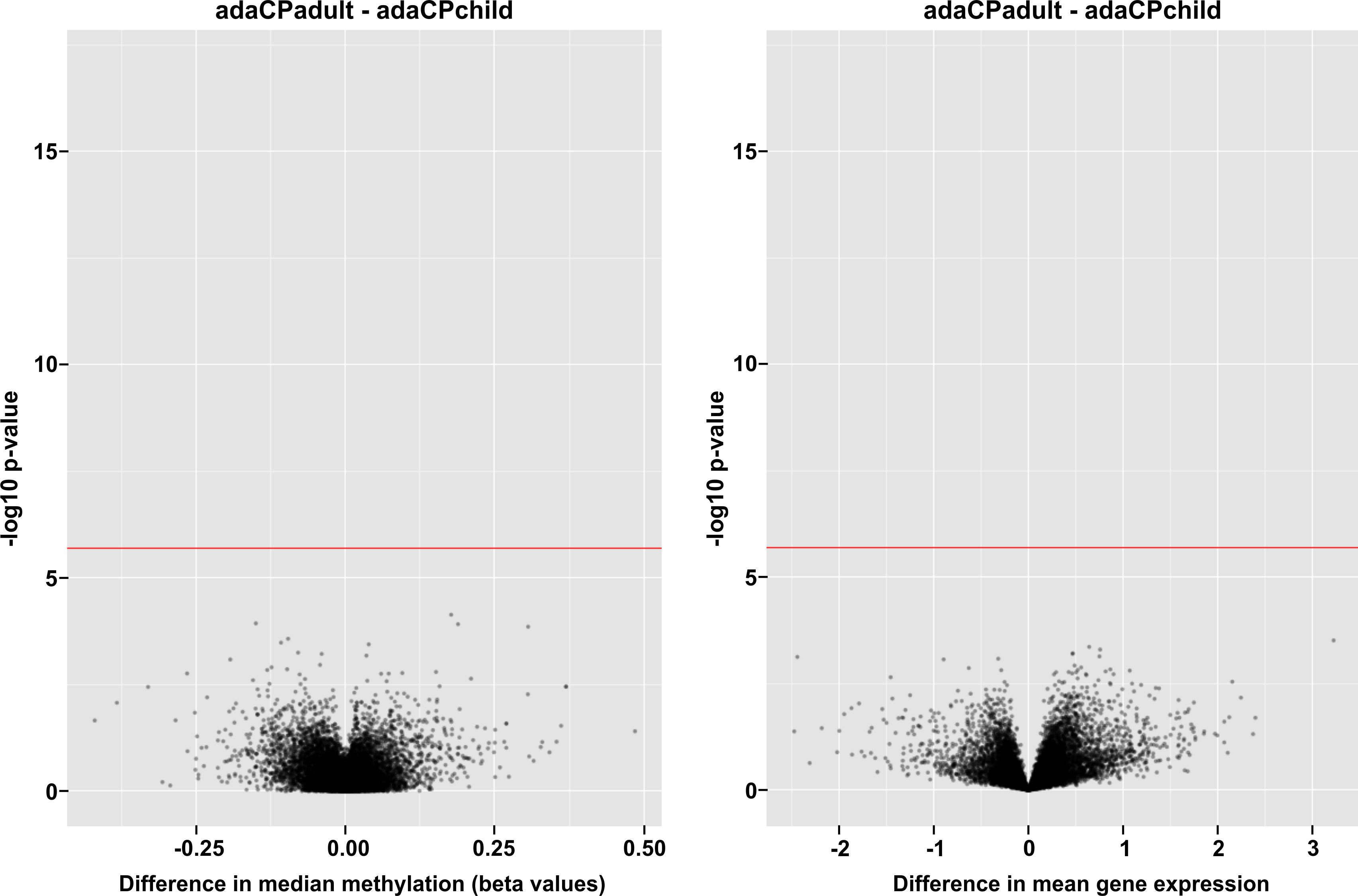

Supplement: Additional file 3: Figure S2. — Pediatric and adult adaCP do not have different methylation or gene expression signatures. Volcano plot (a) showing the difference in median methylation of genes between pediatric (n = 7) and adult (n = 10) adaCP samples on the x-axis and the –log10 transformed p-values of the corresponding t-test results on the y-axis. The volcano plot on the right hand side (b) shows the difference in mean gene expression of genes between pediatric and adult adaCP samples on the x-axis and corresponding –log10 transformed t-test p-values on the y-axis. No gene was found to be significantly differentially methylated or differentially expressed. (JPG 338 kb) [file 40478_2016_287_MOESM3_ESM.jpg]
