# Supplementary material for: Adamantinomatous and papillary craniopharyngiomas are characterized by distinct epigenomic as well as mutational and transcriptomic profiles
Source: Acta Neuropathol Commun. 2016 Feb 29;4:20. doi: 10.1186/s40478-016-0287-6 (PMC4770705; doi:10.1186/s40478-016-0287-6)
Supplement: Additional file 2: Table S1. — Single Affymetrix U133 Plus2.0 expression array data of all adaCP and papCP analysed, given in log2 expression units. (DOC 22.6 kb) [file 40478_2016_287_MOESM2_ESM.doc]

**Supplemental Tab.1**

| **case** | ***LEF1*** | ***AXIN2*** | ***PTCH1*** | ***GLI2*** | ***SHH*** | ***CLDN1*** | ***PROM1*** | ***CD44*** | ***TNC*** | ***MAP2*** |
| --- | --- | --- | --- | --- | --- | --- | --- | --- | --- | --- |
| **ada6** | 8.09 | 8.71 | 10.6 | 7.44 | 4.93 | 9.54 | 6.58 | 9.65 | 11.54 | 9.47 |
| **ada11** | 8.95 | 9 | 11 | 6.87 | 8.97 | 10.79 | 10.21 | 10.05 | 6.8 | 9.3 |
| **ada16** | 8.49 | 9.56 | 11.04 | 7.53 | 7.48 | 9.19 | 6.91 | 10.43 | 9.88 | 9.65 |
| **ada19** | 8.69 | 8.86 | 10.06 | 6.28 | 10.68 | 10.28 | 7.55 | 10.58 | 9.09 | 10.4 |
| **ada21** | 7.86 | 9.11 | 10.67 | 7.48 | 5.55 | 9.24 | 7.18 | 9.09 | 10.12 | 9.13 |
| **ada29** | 8.52 | 9.37 | 10.99 | 7.53 | 6.65 | 8.76 | 6.85 | 11.2 | 9.7 | 8.38 |
| **ada30** | 8.81 | 9.61 | 10.76 | 7.67 | 8.24 | 9.42 | 7.73 | 10.48 | 9.03 | 9.17 |
| **ada31** | 7.94 | 8.24 | 10.81 | 7.76 | 7.44 | 10.76 | 6.74 | 9.44 | 9.04 | 10.05 |
| **ada32** | 8.8 | 9.65 | 10.39 | 6.93 | 6.84 | 9.71 | 7.89 | 10.99 | 9.89 | 10.45 |
| **ada33** | 9.99 | 9.33 | 9.91 | 7.23 | 5.71 | 8.8 | 8.05 | 10.67 | 10.63 | 9.33 |
| **ada37** | 8.38 | 9.35 | 10.9 | 7.52 | 7.31 | 9.84 | 7.19 | 10.09 | 10.82 | 9.34 |
| **ada48** | 9.1 | 9.73 | 11.21 | 7.72 | 6.93 | 8.58 | 6.64 | 9.59 | 10.73 | 9.27 |
| **ada53** | 8.67 | 7.75 | 8.78 | 6.96 | 5.68 | 9.85 | 6.56 | 10.9 | 10.69 | 8.65 |
| **ada54** | 9.09 | 9.92 | 11.1 | 7.04 | 7.47 | 10.44 | 8.51 | 9.22 | 8.39 | 9.91 |
| **ada56** | 7.93 | 9.15 | 11.31 | 7.43 | 8.99 | 11.25 | 6.55 | 10.18 | 7.79 | 9.9 |
| **ada58** | 8.46 | 9.23 | 10.44 | 7.69 | 5.91 | 8.48 | 6.28 | 10.53 | 11.53 | 9.62 |
| **ada59** | 9.6 | 10.18 | 10.61 | 7.42 | 5.99 | 9.67 | 9.16 | 10.46 | 9.05 | 9.98 |
| **ada70** | 8.51 | 9.54 | 10.85 | 7.52 | 8.49 | 10.06 | 7.06 | 10.32 | 8.41 | 9.83 |
| **pap4** | 5.57 | 5.2 | 6.85 | 4.73 | 2.54 | 12.25 | 5.67 | 11.56 | 5.92 | 6.78 |
| **pap7** | 5.56 | 5.2 | 6.51 | 5.13 | 2.29 | 12.51 | 3.23 | 11.51 | 4.98 | 6.74 |
| **pap8** | 4.77 | 5.74 | 7.35 | 5.42 | 4.76 | 12.11 | 4.98 | 11.62 | 6.55 | 7.27 |
| **pap9** | 6.17 | 5.52 | 7.32 | 4.98 | 2.54 | 12.64 | 4.48 | 11.68 | 5.33 | 6.57 |
| **pap11** | 5.41 | 5.54 | 7.03 | 5.59 | 4.11 | 12.52 | 4.41 | 11.38 | 4.91 | 7.21 |
| **pap13** | 7.25 | 5.62 | 6.82 | 5.37 | 3.32 | 12.45 | 3.96 | 11.55 | 7.71 | 8.1 |
| **pap16** | 5.6 | 5.33 | 6.64 | 5.47 | 2.61 | 12.79 | 4.02 | 10.37 | 5.89 | 4.82 |
| **pap18** | 6.5 | 6.69 | 7.86 | 5.4 | 5.19 | 11.92 | 4.73 | 11.69 | 6.92 | 7.72 |
| **pap19** | 6.1 | 5.59 | 7.21 | 4.39 | 4.96 | 12.34 | 3.71 | 11.76 | 4.59 | 5.55 |
| **pap20** | 5.5 | 4.52 | 5.53 | 5.63 | 4.42 | 12.2 | 2.68 | 11.86 | 7.9 | 5.76 |
